# Absorption of the Testes from Metastasis of Mumps

**Published:** 1850-10

**Authors:** Columbus Hixson

**Affiliations:** Student of Medicine, Middletown, Guernsey County, Ohio


*Absorption of the Testes from Metastasis of Mumps.* By Columbus Hixson, Student of Medicine, Middletown, Guernsey County, Ohio.

A. G., aged 25, in April, 1842, was attacked with mumps, which at that time were prevailing in an epidemic form in the neighborhood. The family physician was called, who prescribed the usual antiphlogistic treatment. In the course of three weeks the patient had so far recovered as to be able to walk about his chamber. The swelling of the parotid glands had entirely disappeared, and the patient thinking himself convalescent, made a visit upon horseback to the village about one and a half miles distant; and returned in about 3 hours, with slight sensations of pain in the testicles, which in the course of a few hours became so severe as to confine him to his bed. The physician was again called, who prescribed fomentations, a suspensory bandage, and the unguentum iodini comp. to the scrotum, and rubefacients to the parotids. Under this treatment the patient speedily recovered, the swelling was dispersed and he has ever since enjoyed excellent general health. Shortly after this occurrence, he noticed small tumors forming in the substance of the scrotum, which, at the present time are very numerous, but most abundant on the left side of the scrotum, and which at times run on to suppuration, and when punctured, some emit a fluid resembling healthy pus, while others discharged a fluid of the consistence of paste. The scrotum has also been attended with itching sensations, which at times is almost intolerable.

Shortly after his convalescence from the mumps, our patient noticed that his testicles were becoming smaller, which caused a great deal of uneasiness. Several medical gentlemen were consulted, but none gave him any encouragement respecting a cure. At last he disclosed the case to the writer, who, after examination, determined to report the case, hoping that it might elicit from the pen of some one an investigation. The testicles of this individual, at the present time, are not more than one third their original size, the right one is considerably smaller than the left.

From this history of the case, which is a correct one, there appears to have been a departure from the ordinary phenomena observed in the course of this disease. So far as the writer is aware it is the doctrine of pathologists, *that the testicles are liable to become*

*implicated at the time of the swelling leaving the parotid glands.* In this case it appears from the patient's own acknowledgments that *swelling or inflammation had entirely left its original seat,* and he had so far recovered as to consider himself convalescent. In the opinion of the writer, the tumors present in the scrotum, are but a morbid condition of the sebaceous glands of that part. Should this article meet the eye of any medical gentleman, who feels himself competent to the task, it is hoped that the treatment most likely to succeed will be pointed out.

